# Music Training and Education Slow the Deterioration of Music Perception Produced by Presbycusis in the Elderly

**DOI:** 10.3389/fnagi.2017.00149

**Published:** 2017-05-19

**Authors:** Felipe N. Moreno-Gómez, Guillermo Véliz, Marcos Rojas, Cristián Martínez, Rubén Olmedo, Felipe Panussis, Alexies Dagnino-Subiabre, Carolina Delgado, Paul H. Delano

**Affiliations:** ^1^Laboratorio de Neurobiología de la Audición, Programa de Fisiología y Biofísica, Instituto de Ciencias Biomédicas (ICBM), Facultad de Medicina, Universidad de ChileSantiago, Chile; ^2^Auditory and Cognition Center, AUCOSantiago, Chile; ^3^Departamento de Biología y Química, Facultad de Ciencias Básicas, Universidad Católica del MauleTalca, Chile; ^4^Departamento de Otorrinolaringología, Hospital Clínico de la Universidad de ChileSantiago, Chile; ^5^Laboratorio de Neurobiología del Stress, Centro de Neurobiología y Plasticidad Cerebral (CNPC), Instituto de Fisiología, Facultad de Ciencias, Universidad de ValparaísoValparaíso, Chile; ^6^Departamento Neurología y Neurocirugía, Hospital Clínico de la Universidad de ChileSantiago, Chile

**Keywords:** music, music perception, aging, elderly, presbycusis, amusia, MBEA

## Abstract

The perception of music depends on the normal function of the peripheral and central auditory system. Aged subjects without hearing loss have altered music perception, including pitch and temporal features. Presbycusis or age-related hearing loss is a frequent condition in elderly people, produced by neurodegenerative processes that affect the cochlear receptor cells and brain circuits involved in auditory perception. Clinically, presbycusis patients have bilateral high-frequency hearing loss and deteriorated speech intelligibility. Music impairments in presbycusis subjects can be attributed to the normal aging processes and to presbycusis neuropathological changes. However, whether presbycusis further impairs music perception remains controversial. Here, we developed a computerized version of the Montreal battery of evaluation of amusia (MBEA) and assessed music perception in 175 Chilean adults aged between 18 and 90 years without hearing complaints and in symptomatic presbycusis patients. We give normative data for MBEA performance in a Latin-American population, showing age and educational effects. In addition, we found that symptomatic presbycusis was the most relevant factor determining global MBEA accuracy in aged subjects. Moreover, we show that melodic impairments in presbycusis individuals were diminished by music training, while the performance in temporal tasks were affected by the educational level and music training. We conclude that music training and education are important factors as they can slow the deterioration of music perception produced by age-related hearing loss.

## Introduction

The perception of music depends on the normal function of the auditory system, including cochlear receptor cells, auditory nerve neurons and the central auditory pathways (Särkämö et al., 2013; Wipe et al., 2013; Theunissen and Elie, 2014). In addition to the auditory system, music stimuli recruit other regions of the brain like emotion, memory, reward and motor circuits (Peretz and Zatorre, 2005; Groussard et al., 2010; Zatorre and Salimpoor, 2013). Therefore, in a neurobiological context, music perception can be thought as a complex brain function including sensorimotor and cognitive networks (Särkämö et al., 2013). Importantly, these brain circuits are affected by age-related neurodegenerative processes, causing hearing and cognitive impairments (Panza et al., 2015).

The acoustical features of music stimuli comprise (i) pitch, (ii) temporal and (iii) timbral components (Janata, 2015). The frequency content of music stimuli, including the fundamental frequency and its harmonics constitute the bases of musical pitch. The temporal properties of a sequence of acoustical stimuli are the bases of musical rhythm and meter, while the timbral dimension allows recognition of auditory objects. These acoustic features have a counterpart in brain processing, as empirical evidence indicate that distinctive brain areas are active when processing different musical components (Stewart et al., 2006; Janata, 2015). For instance, Liégeois-Chauvel et al. (1998) found that an intact posterior superior temporal gyrus (STG) is fundamental for melodic processing, while the anterior STG is important for temporal processing.

Aging affects music perception, including pitch and temporal components. For example, elderly subjects without hearing complaints and with normal audiometric thresholds [≤25 dB hearing level (HL) between 0.5 and 4 kHz] have lower performance in frequency discrimination (Clinard et al., 2010) and modulation tasks (He et al., 2007). In addition, aged subjects with normal hearing have reduced brainstem responses to consonant/dissonant two-note cords (Bones and Plack, 2015). Temporal processing is also impaired in aged individuals, as evidenced by psychoacoustic (Gordon-Salant et al., 2011) and electrophysiological (Harris et al., 2012) assessment of gaps in noise tasks. Together, these studies show the presence of age-related perceptual and physiological acoustical impairments, in the absence of symptomatic hearing loss.

Age-related hearing loss or presbycusis is a frequent condition in elderly subjects, with an estimated global prevalence of around 360 million people (WHO, 2014). Presbycusis is produced by age-related neurodegenerative processes that affect the cochlear receptor cells, auditory nerve neurons and brain circuits involved in auditory perception (Gates and Mills, 2005; Frisina, 2009; Lin et al., 2014; Ouda et al., 2015). Symptomatic presbycusis patients (e.g., hearing loss >35 dB HL, subjects requiring hearing aids) present bilateral high-frequency hearing loss, and deteriorated speech intelligibility, especially in the presence of background noise or reverberation (Mazelová et al., 2003; Van Eyken et al., 2007). Moreover, age-related hearing loss has been proposed as a risk factor to develop age-related cognitive impairment (Lin and Albert, 2014; Wayne and Johnsrude, 2015). Whether presbycusis is an additional factor to age that deteriorates pitch and temporal perception is controversial. For instance, similar aging effects in normal hearing and in presbycusis patients have been obtained in the discrimination of tone sequences (Fitzgibbons and Gordon-Salant, 2015), while greater disability for unaccented and accented monosyllabic words has been observed in presbycusis subjects (Gordon-Salant et al., 2015). Regarding auditory temporal resolution, Humes et al. (2010) found that the age-related impairments in gap detection were mediated by hearing loss, while Ozmeral et al. (2016) found that after adjusting by hearing sensitivity, aging was the most important factor determining gap detection. It is important to note that the majority of pitch and temporal perception studies in aged subjects were performed in relatively mild presbycusis patients with auditory thresholds better than 40 dB HL, which do not have explicit hearing complaints, but could have alterations in central auditory processing (Humes et al., 2010; Fitzgibbons and Gordon-Salant, 2015; Gordon-Salant et al., 2015; Ozmeral et al., 2016).

The principal aim of this study was to evaluate whether presbycusis is an additional factor to aging that negatively affects music perception, and whether this was influenced by educational level and music training. We developed a computerized version of the Montreal battery of evaluation of amusia (MBEA, Peretz et al., 2003) and evaluated music perception in Chilean adults aged between 18 and 90 years without hearing complaints and in symptomatic presbycusis patients.

## Materials and Methods

### Subjects

A total of 175 subjects were recruited, including 133 individuals between 18 and 85 years as controls, and 42 subjects with symptomatic age-related hearing loss (presbycusis) between 64 and 90 years. Symptomatic presbycusis patients (hearing loss between 0.5 and 4 kHz >35 dB HL) were prospectively recruited from patients above 60 years that consulted for hearing loss complaints at the Otolaryngology Department of the Clinical Hospital of the University of Chile as part of the Chilean government program^1^ to fit hearing aids in presbycusis patients. Controls that gave a self-report of no feeling of hearing loss (Sindhusake et al., 2001), and had no history of otological and audiological diseases were recruited from relatives of consulting presbycusis patients and from University staff and students. The screening hearing handicap inventory for the elderly (HHIE-S) was applied in controls and presbycusis subjects older than 59 years. The HHIE-S has been validated for Spanish speaking population (Lichtenstein and Hazuda, 1998) and measures hearing complaints in daily life. Following suggestions given by Lichtenstein and Hazuda (1998) for Spanish speaking population, possible control subjects with more than 10 points in the HHIE-S were excluded from this study. Presbycusis was confirmed using audiometric thresholds that evidenced bilateral and symmetric hearing loss greater than 35 dB HL in pure tone averages (PTA) between 0.5 and 4 kHz. Patients with middle ear pathology, evidenced by examination of the tympanic membrane or by audiological tests (e.g., conductive hearing loss or flat middle ear compliance) were excluded from this study. All volunteers were Chileans, used Spanish as their native language, and had no clinical history of neurological and psychiatric disorders. All procedures were approved by the scientific ethics committee of the Clinical Hospital of the University of Chile. All subjects gave written informed consent in accordance with the Declaration of Helsinki.

### Experimental Procedure

#### Audiometry

Air conduction thresholds of pure tones at 0.25, 0.5, 1, 2, 3, 4, 6, and 8 kHz were evaluated and stored as hearing levels decibels in the 42 presbycusis subjects. Measurements were performed in audiometric sound-proof rooms using TDH-39 headphones and a calibrated audiometer (ANSI S3.6-2010). PTAs were calculated using 0.5, 1, 2, and 4 kHz thresholds. Speech discrimination was assessed at a comfortable level between 30 and 40 dB above PTA thresholds, and computed as percentage of discrimination using a total of 25 disyllabic words. As we have two perceptual measures (ears) per subject, for analysis purposes, the best ear of each audiometric variable was included in the analysis.

#### Montreal Battery for the Evaluation of Amusia

The MBEA was developed by Peretz et al. (2003) to detect subjects with music perception impairments or amusia, and has been widely used to detect music perception deficits (amusia) in pitch, temporal and memory dimensions (Hyde et al., 2006; Gosselin et al., 2009; Albouy et al., 2013; Kalathottukaren et al., 2015). An automatized version of the MBEA was developed in C programming language (LabWindows CVI 6.0 from National Instruments) and used with a graphic user interface that allowed subjects to respond with a mouse click. The acoustic stimuli used in the MBEA were digitized at 16 bit and sampled at 44.1 kHz. These stimuli were presented using a 20 to 20,000 Hz free-field speaker at a comfortable intensity (30 to 40 dB above PTA thresholds) in a sound-attenuating room. The MBEA is organized in six different tasks with 30 trials each, in which subjects have to respond in a two choice paradigm. The first three tasks are designed to measure melodic components of music, including pitch scale (T1), contour (T2) and interval (T3). The fourth and fifth tasks assess the temporal dimension, including rhythm (T4) and meter (T5), while the sixth task measure musical memory (T6) (Peretz et al., 2003). To assure volunteer comprehension of the tasks, each one was preceded by two or four example trials. In addition, to guarantee volunteers attention and motivation, catch trials (which are easily differentiated, as they vary in several acoustic dimensions) were presented between the trials. Subjects that failed to detect catch trials were excluded from this work. In addition to the MBEA performance, epidemiological data of volunteers, including age, sex, years of education, years of music training and handedness were stored. For analyses purposes in the multifactorial models music training was considered as a binary variable (yes/no), considering a “yes” response, at least 1 year of music training.

### Statistical Analysis

First, we analyzed MBEA global and tasks (T1–T6) performances (the number of correct responses and accuracy [100^∗^number of correct responses/(correct + incorrect responses)]) in the three studied groups: (1) controls between 18 and 60 years (*n* = 84), (2) controls aged > 60 years (*n* = 49) and (3) presbycusis subjects > 60 years (*n* = 42). The analysis of the two groups of control subjects aged between 18 and 85 years (*n* = 133) allowed us to calculate reference values, including means, and cut-off values for global and tasks MBEA performances. Normal distribution of data was evaluated using Shapiro–Wilk tests, and differences between groups were evaluated with Kruskal–Wallis and Dunn *post hoc* tests. Differences between frequency counts of the histogram distributions were evaluated with *X*^2^-tests. Fisher exact test was used to evaluate the numbers of amusic subjects in the three different groups. Descriptive statistics were performed using the Systat software Sigmaplot v12.5. Within all statistical tests *p-*values < 0.05 were considered as significant.

Next, to study possible relations of MBEA performance with demographic and audiological data in the aged population, we built generalized linear models (GLMs) with R programming language (R version 3.2.1, R Core Team, 2015) using two datasets and excluding controls <61 years. The first dataset included the 49 control individuals aged more than 60 years and the 42 patients with symptomatic presbycusis (*n* = 91). This dataset was used to evaluate the effects of presbycusis, age, sex, years of formal education and musical training. The second dataset included only presbycusis individuals (*n* = 42). In this case, we evaluated the effects of education, musical training and different audiological measures, including PTA thresholds, the percentage of word discrimination and HHIE-S scores.

Models were fitted using a binomial family and a logit link, however, a quasibinomial family was used in the presence of overdispersion. We evaluated the effect of each factor by separate and also controlling for the effect of the other factors. In this last case, we first fitted a full model that was simplified to obtain the minimal adequate model. The full model included all the main effects and their paired interactions. Model simplification was performed using *X*^2^-tests or *F*-tests depending whether a binomial or a quasibinomial family was used, respectively. The less significant factor was removed each time. We first removed the interactions and then the main effects. If an interaction was significant but not one of the main effects included in the interaction, the factor was not removed from the model. Model simplification stopped when a significant difference between the tested models occurred (*p*-value < 0.05), allowing obtaining the minimal adequate model. We checked if the model showed overdispersion, and in that case the model was refitted using a quasibinomial family. The significance of each factor was obtained with a Type-III analysis of variance (Wald-test for binomial family and *F*-test for quasibinomial family) using the R library “car” (Fox and Weisberg, 2010). While presbycusis, sex and musical training were included as categorical predictors, the other variables where included as continuous predictors. In the case of meter-task analyses (T5) an influential outlier having a low score was removed from the model.

## Results

**FIGURE 1:**
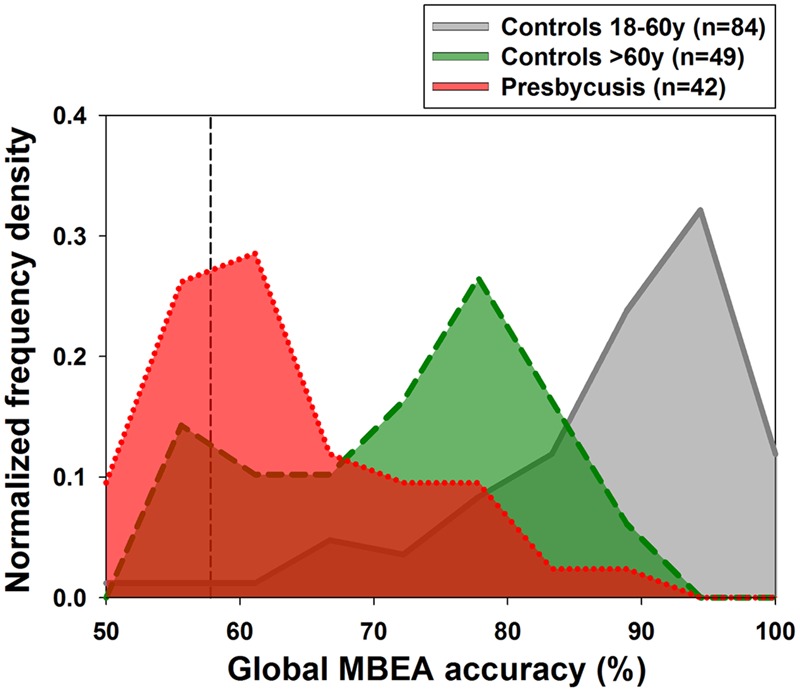
**Normalized histograms of global montreal battery of evaluation of amusia (MBEA) accuracy in the three studied groups: (i) gray plot displays the 18–60 years group (*n* = 84), (ii) green plot >60 years controls (*n* = 49), and the red plot (iii) presbycusis subjects (*n* = 42).** The segmented vertical line represents (57.8%) the mean minus two standard deviations of control subjects aged between 18 and 85 years used to detect amusic subjects. Note the presence of three peaks at different values for each studied group.

A total of 175 subjects between 18 and 90 years successfully completed the MBEA. Data were analyzed separately into three groups: (i) controls between 18 and 60 years (*n* = 84), (ii) controls older than 60 years (*n* = 49) and (iii) presbycusis subjects (*n* = 42). A summary of age, sex, educational level and music training of the three groups is shown in **Table 1**. Descriptive statistics showing means, standard deviations and cut-off scores obtained from the two groups of controls (*n* = 133) are shown in **Table 2**. **Table 2** allows comparison with original data published by Peretz et al. (2003), and the generation of our own population based cut-off score of global MBEA accuracy at 57.8% using the mean minus two standard deviations of all control subjects aged between 18 and 85 years. Using this criterion, the fraction of amusic subjects in the three studied groups were significantly different (Fisher exact test, *p* < 0.001), as one out of 84 subjects (1.2%) of the controls between 18 and 60 years, five out of 49 (10.2%) of the aged controls (>60 years), and nine out of 42 (21.4%) of presbycusis patients can be classified as amusic subjects. Similarly, there were significant separations between the frequency counts of the histogram distributions of global MBEA accuracy in the three evaluated groups [**Figure 1**, *X^2^*_(18)_ = 118.55, *p* < 0.001].

**Table 1:** Epidemiological data of the 175 subjects that performed the montreal battery of evaluation of amusia (MBEA).

	Age (mean years, *SD*)	Education (mean years, *SD*)	Male/female number	Music training n/total (%)	Music training years (mean years, *SD*)	Average years of music training in those with at least 1 year (mean years, *SD*) (n)	Amusic n/total (%)
Controls (18–60 years) *N* = 84	34.7 ± 16.0	16.0 ± 3.2	41/43	32/84 (38.1%)	2.47 ± 5.78	6.47 ± 7.92 (32)	1/84 (1.2%)
Controls >60 years *N* = 49	72.7 ± 6.4	9.9 ± 5.5	20/29	11/49 (22.4%)	1.29 ± 3.80	5.72 ± 6.33 (*n* = 11)	5/49 (10.2%)
Presbycusis *N* = 42	77.7 ± 6.3	9.9 ± 4.8	19/23	11/42 (26.2%)	1.05 ± 3.20	4.00 ± 5.50 (*n* = 11)	9/42 (21.4%)
Total N = 175	55.7 ± 22.9	12.9 ± 5.3	80/95	54/175 (30.9%)	1.79 ± 4.77	5.82 ± 7.14 (*n* = 54)	15/175 (8.6%)

**Table 2:** Descriptive statistics on the 30 experimental trials for each test of the MBEA obtained by 133 control subjects between 18 and 85 years.

	Scale (T1)	Contour (T2)	Interval (T3)	Rhythm (T4)	Meter (T5)	Memory (T6)	Average
Mean	24.4	24.1	22.4	25.5	25.2	24.1	24.3
SD	4.3	4.6	5.1	3.9	4.1	4.7	4.5
Median	26.0	26.0	23.0	27.0	26.0	25.0	25.5
% subjects with perfect score	7	11	7	15	16	15	11.8
Cut-off score (5% confidence interval)	15.7	15.0	14.0	16.0	17.7	16.0	15.7
Number and percentage of subjects below cut-off score	6 (4.5%)	2 (1.5%)	4 (3.0%)	4 (3.0%)	6 (4.5%)	4 (3.0%)	4.3 (3.2%)

### Performance in the Six Tasks of the MBEA

**FIGURE 2:**
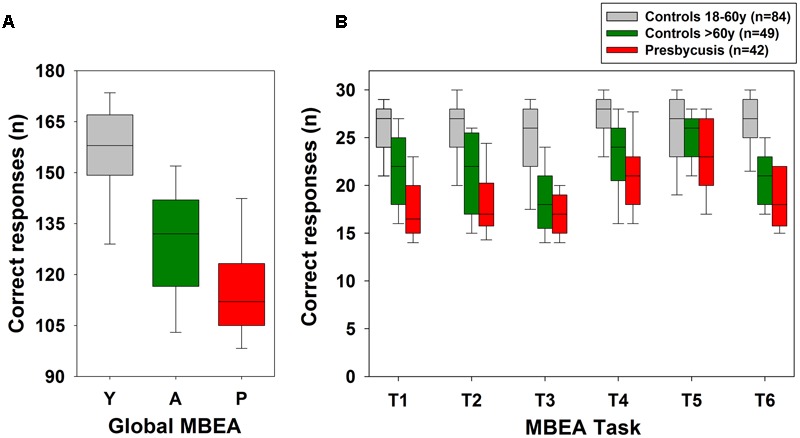
**Montreal battery of evaluation of amusia correct responses in the three different groups: (i) 18–60 years group (gray box-plots), (ii) controls >60 years (green box-plots) and in (iii) symptomatic presbycusis patients (red box-plots).** Correct responses in **(A)** Global and in **(B)** the six tasks of the MBEA. Significant differences between aged and young subjects (18–60 years) were obtained in global and in all tasks, except meter task (T5). Significant differences between aged controls and presbycusis subjects were obtained in global and task T1. (Y: young controls; A: aged controls; P: presbycusis patients).

**Figure 2** shows box-plots of correct responses in the global MBEA and in the six tasks (T1–T6) of the MBEA in three studied groups. Correct responses in the global scores and in the six tasks of the MBEA were not normally distributed (Shapiro–Wilk, *p* < 0.05). A Kruskal–Wallis analysis followed by a Dunn *post hoc* test showed significant differences in global MBEA correct responses [*H*_(2)_ = 87.987, *p* = 0.001] between the three studied groups (**Figure 2A**). Similarly, correct responses during task T1 were significantly different between the three studied groups [Kruskal–Wallis, *H*_(2)_ = 82.793, *p* < 0.001, Dunn *post hoc* test]. MBEA tasks T2, T3, T4, and T6 have significant differences between the groups of controls ≤60 years compared to aged controls and presbycusis subjects, but no differences between aged controls and presbycusis [T2: Kruskal–Wallis, *H*_(2)_ = 72.606, *p* < 0.001; T3: *H*_(2)_ = 73.739, *p* < 0.001; T4: *H*_(2)_ = 61.236, *p* < 0.001; T6: *H*_(2)_ = 86.829, *p* < 0.001, Dunn *post hoc* tests]. Regarding task T5, the only significant difference was obtained between controls ≤60 years and presbycusis subjects [Kruskal–Wallis, *H*(2) = 8.652, *p* = 0.013, Dunn *post hoc* test], while there was no significant difference between controls ≤60 years and aged controls.

### Factors Contributing to MBEA Performance

**FIGURE 3:**
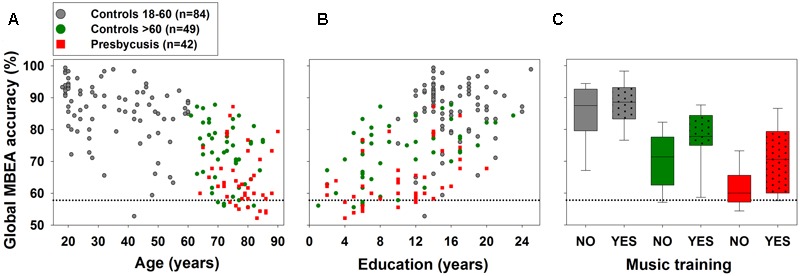
**Aging, education, music training and presbycusis are important factors for global MBEA accuracy.** Presbycusis patients are depicted in red squares and box-plots, while 18–60 years and >60 years controls in gray and green symbols and box-plots correspondingly. The dotted horizontal lines represent the 57.8% cut-off score of global MBEA accuracy. **(A,B)** Note that aging and lower educational level correlate with worse global MBEA accuracy. **(C)** Box-plots show median and interquartile range of subjects with music education (illustrated by punctate patterns) compared to no music education in the three studied groups. Notice that music training enhances global MBEA accuracy in aged and presbycusis subjects.

We studied possible factors contributing to individual and group MBEA performance. **Figure 3** shows individual global MBEA accuracy data plotted with corresponding age, years of education, and group differences between subjects with or without music training. GLMs were used to evaluate a possible dependence of global MBEA accuracy on age, education, sex, music training, and symptomatic presbycusis in subjects aged 60 or more years (*n* = 91). GLMs fitted to test the effect of each factor separately showed a significant dependence of global MBEA performance on age [*F*_(1,89)_ = 11.69, *p* = 0.0009502], education [*F*_(1,89)_ = 24.745, *p* = 3.165^∗^10^-6^], music training [*F*_(1,89)_ = 8.796, *p* = 0.003875], and presbycusis [*F*_(1,89)_ = 16.678, *p* = 9.66^∗^10^-5^] while no significant effect was found for sex [*F*_(1,89)_ = 1.829, *p* = 0.1797]. The minimal adequate models fitted to evaluate the effect of each factor while controlling for the effects of the other factors on global MBEA accuracy suggest that the most relevant factors were presbycusis [*F*_(1,85)_ = 26.9284, *p* = 1.416^∗^10^-6^], education [*F*_(1,85)_ = 26.4913, *p* = 1.682^∗^10^-6^] and the interaction between musical education and sex [*F*_(1,85)_ = 6.2886, *p* = 0.01405]. The results of the minimal adequate models of the effects of the six tasks of the MBEA indicated that presbycusis had a significant effect on tasks T1, T2, T4, T5, and T6, while education was significant in tasks T1, T2, T3, T4, and T5, and music training in T1, T2, T3, T5, and T6. **Table 3** shows a summary of the statistics values of the minimal adequate models for the performances in the global and six tasks of the MBEA in aged (>60 years) and presbycusis subjects.

**Table 3:** Minimal adequate models obtained for MBEA performance using the dataset including subjects >60 years (*n* = 49) and presbycusis patients (*n* = 42).

MBEA task	Significant factors	Freedom degrees	*F*-value	*P*-value
Global	PRESB	1, 85	26.928	**<0.001**
	EDU	1, 85	26.491	**<0.001**
	MUSTRAIN	1, 85	0.011	0.917
	SEX	1, 85	0.152	0.698
	MUSTRAIN:SEX	1, 85	6.289	**0.014**

T1	PRESB	1, 87	28.514	**<0.001**
	EDU	1, 87	6.542	**0.012**
	MUSTRAIN	1, 87	4.141	**0.045**

T2	PRESB	1, 82	17.497	**<0.001**
	AGE	1, 82	1.832	0.180
	EDU	1, 82	24.037	**<0.001**
	MUSTRAIN	1, 82	5.165	**0.026**
	SEX	1, 82	1.907	0.171
	PRESB:MUSTRAIN	1, 82	5.744	**0.019**
	AGE:MUSTRAIN	1, 82	6.179	**0.015**
	MUSTRAIN:SEX	1, 82	7.740	**0.007**

T3	AGE	1, 86	1.077	0.302
	EDU	1, 86	7.777	**0.007**
	MUSTRAIN	1, 86	4.975	**0.028**
	AGE:EDU	1, 86	6.373	**0.013**

T4	PRESB	1, 87	7.023	**0.010**
	EDU	1, 87	39.563	**<0.001**

T5	PRESB	1, 86	7.757	**0.007**
	EDU	1, 86	6.075	**0.016**
	MUSTRAIN	1, 86	5.012	**0.028**

T6	PRESB	1, 87	10.492	**0.002**
	MUSTRAIN	1, 87	6.260	**0.014**
	SEX	1, 87	4.687	**0.033**

*The total individuals for modeling were 91. PRESB, presbycusis; EDU, years of formal education; MUSTRAIN, musical training; SEX, sex; AGE, age. Significant *p*-values are in bold.*

### Audiological Factors and MBEA Performance

**FIGURE 4:**
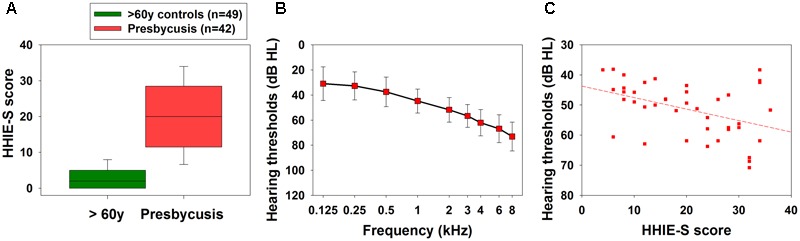
**Hearing handicap inventory for the elderly (HHIE-S) in aged subjects (>60 years) and mean hearing thresholds in presbycusis patients. (A)** A significant difference in hearing complaints between these groups were found in HHIE scores. **(B)** Average audiometric thresholds, obtained between 0.5 and 8 kHz in presbycusis patients (mean ± SEM). **(C)** Significant correlation between individual HHIE-S and PTA (average between 0.5 and 4 kHz) [*r*_(42)_ = 0.392, *p* = 0.01, Spearman].

**Figure 4** shows HHIE-S scores and mean audiometric thresholds in frequencies between 0.25 and 8 kHz in presbycusis subjects. We found a significant correlation between hearing complaints (HHIE-S) and PTA audiometric thresholds [**Figure 4C**, Spearman, *R*_(42)_ = 0.392, *p* = 0.01]. To determine possible audiological factors contributing to the MBEA performance in presbycusis subjects (*n* = 42), GLMs were fitted considering audiometric thresholds, speech discriminations and HHIE-S scores in addition to years of education and musical training as potential explanatory variables. The minimal adequate model for global MBEA accuracy in presbycusis patients included education, musical training and the interaction between musical training and HHIE-S scores; however, this last factor was not significant as a main effect (**Table 4**). Regarding the six tasks of the MBEA, musical training was included in the minimal adequate models of the three melodic tasks, one temporal task and in the memory task (T1, T2, T3, T5, and T6), while education was included in both temporal tasks (T4 and T5). HHIE-S was a significant main effect factor in T5 and was included in significant interactions in T2, T3, T5, and T6. Audiological measures (PTA thresholds and speech discrimination) were significant factors included in the minimal adequate model for the accuracy of MBEA meter and memory task (T5 and T6), and PTA appears in a significant interaction in T2. A summary of factors included in minimal adequate models for MBEA performance in presbycusis subjects is shown in **Table 4**. When tested separately, audiological variables showed non-significant effects on global and specific tasks (T1–T6) of MBEA performance (data not shown).

**Table 4:** Minimal adequate models obtained for MBEA performance using the dataset including only presbycusis patients (*n* = 42).

MBEA task	Significant factors	Freedom degrees	*F*/*X*^2^-value	*P*-value
Global	HHIE	1, 37	0.007	0.933
	EDU	1, 37	7.691	**0.009**
	MUSTRAIN	1, 37	14.868	**0.001**
	HHIE:MUSTRAIN	1, 37	10.556	**0.003**

T1	MUSTRAIN	1, 40	4.403	**0.042**

T2	PTA	1, 36	1.906	0.176
	HHIE	1, 36	0.815	0.373
	MUSTRAIN	1, 36	5.172	**0.029**
	PTA:MUSTRAIN	1, 36	18.340	**<0.001**
	HHIE:MUSTRAIN	1, 36	30.439	**<0.001**

T3	HHIE	1	0.076	0.782
	MUSTRAIN	1	9.753	**0.002**
	HHIE:MUSTRAIN	1	7.321	**0.007**

T4	EDU	1, 40	10.861	**0.002**

T5	PTA	1, 32	11.316	**0.002**
	HHIE	1, 32	5.605	**0.024**
	DISCRI	1, 32	7.253	**0.011**
	EDU	1, 32	8.083	**0.008**
	MUSTRAIN	1, 32	22.574	**<0.001**
	PTA:EDU	1, 32	5.303	**0.028**
	PTA:MUSTRAIN	1, 32	19.797	**<0.001**
	HHIE:DISCRI	1, 32	5.184	**0.030**

T6	PTA	1, 36	8.277	**0.007**
	HHIE	1, 36	3.817	0.059
	DISCRI	1, 36	9.153	**0.005**
	MUSTRAIN	1, 36	8.815	**0.005**
	HHIE:MUSTRAIN	1, 36	5.902	**0.020**

*PTA, pure tone DISCRI: Speech discrimination; EDU, years of formal education; MUSTRAIN, musical training. Significant factors are in bold. In the case of T3, the statistic test corresponds to *X*^*2*^-value. Significant *p-*values are in bold.*

## Discussion

The principal aim of the present work was to determine whether in addition to the normal process of brain aging, presbycusis further impairs music perception, including pitch and temporal components. We found that symptomatic presbycusis is the most relevant factor explaining the observed variation in global MBEA accuracy in aged subjects (>60 years) [*F*_(1,85)_ = 26.93, minimal adequate model, **Table 3**], showing that music perception is more altered in presbycusis patients than in aged controls with no hearing complaints. Moreover, music perception impairments in melodic dimensions (MBEA tasks T1, T2, and T3) in presbycusis individuals were diminished by music training (minimal adequate model, **Table 4**), while the performance in temporal tasks were affected by the educational level and music training.

### Normative MBEA Data on Chilean Population

The MBEA scores obtained in this work allowed us to compute normative data for the Chilean adult population. **Table 2** shows that, except for the rhythm task (T4), in average, our Chilean sample of controls aged between 18 and 85 years had two less correct responses than the original Canadian population used by Peretz et al. (2003). Similarly, our own cut-off scores (with two SD) were lower than those reported in Canadians, United States, and Chinese population (Peretz et al., 2003; Cuddy et al., 2005; Nan et al., 2010; Pfeifer and Hamann, 2015). One important factor to explain these differences is the age of the evaluated subjects, as the Chinese and United States groups were young volunteers (<40 years), while our normative sample included people between 18 and 85 years. Differences with the Canadian group, which included subjects between 14 and 79 years, could be attributed to cultural differences between Canadian and Latin-American cultures. For instance, Paraskevopoulos et al. (2010) found cultural differences, mainly in the rhythm task between the original and a Greek version of the MBEA. In our case, the Chilean population is influenced by western music, but also by music from different indigenous cultures such as Andean cultures in the northern region of Chile, and by Mapuche culture from the southern region, being the latter predominantly monotonic and rhythmic music (Robertson, 2007). Therefore, we propose that MBEA performance differences can be explained by different cultural and educational background of Chilean subjects.

### Aging Effects on MBEA Scores

Aged subjects have lower performance when completing tests aimed to evaluate the perception of different features of sounds. For instance, older individuals have difficulties discriminating different frequencies (Clinard et al., 2010), frequency modulations (He et al., 2007), distinguishing consonant/dissonant two-note cords (Bones and Plack, 2015), determining pitch variations (Russo et al., 2012), recognizing changes in sound sequence presentation (Fitzgibbons and Gordon-Salant, 2001; Fitzgibbons et al., 2006) and the occurrence of gaps between tones (Schneider and Hamstra, 1999). Here, we found that meter perception (T5) is preserved in aged subjects with no hearing complaints (**Figure 2**), while pitch and rhythm perception, and musical memory skills are affected by the normal aging processes. A speculative explanation could arise from the case of a musician with a brain tumor that had a right posterior temporal lesion (Baird et al., 2014). After surgery, he was evaluated with the MBEA, showing that meter perception (T5) was preserved, while melodic, rhythm and memory skills were impaired (Baird et al., 2014). In agreement with Liégeois-Chauvel et al. (1998), Baird et al. (2014) proposed that the cortical region located in the right posterior STG is important for melodic, rhythm, and memory abilities, while the anterior STG is critical for meter perception. On the other hand, Sihvonen et al. (2016) studied 77 patients with post-stroke acquired amusia and found that right hemisphere lesions were the most commonly affected brain regions, including the STG, insula and striatum. In addition, they found that temporal anterior lesions were more frequent in rhythm amusia, while posterior temporal and parietal lesions in pitch amusia. Together, neuroanatomical studies show that the brain regions involved in music perception are lateralized to the right STG, however, more studies are needed to define more precisely specific brain regions involved in pitch and temporal dimensions.

### Presbycusis and Music Perception

Whether there are specific music perception impairments in presbycusis patients is relatively unknown, as only a few studies have evaluated music perception in symptomatic presbycusis patients. For instance, Fitzgibbons and Gordon-Salant (2015) studied the discrimination of intervals within rhythmic tone sequences in aged subjects and in presbycusis patients. These authors found no differences between normal-hearing aged controls (*n* = 13) and presbycusis patients (*n* = 15). However, probably the sample sizes of the evaluated groups were not enough to demonstrate significant differences between aged subjects with normal hearing compared to presbycusis patients. In the present work, we found that presbycusis was the most important factor determining global MBEA accuracy. Specifically, presbycusis was an important factor for melodic (T1 and T2), temporal (T4 and T5), and memory tasks (T6) (**Table 3**).

### Music Training and Education

Because music discrimination of melodic and temporal dimensions can be considered as a cognitive task (Peretz and Zatorre, 2005; Zatorre and Salimpoor, 2013), background experience is an important factor determining performance, constituting an auditory or cognitive reserve (Middleton and Yaffe, 2009; Skoe and Kraus, 2014). For instance, the educational level is a known factor that reduces cognitive decline (Qiu et al., 2001), and a positive relationship between years of education and the performance on auditory processing tests has been found (Murphy et al., 2016). Moreover, although musicians and non-musicians may show similar decays in auditory thresholds with age, musical trained individuals show a better performance during auditory tasks (Zendel and Alain, 2012).

Here, we found that the educational level and music training diminished the alterations observed in music discrimination in presbycusis patients (**Figure 3** and **Table 4**). The mechanisms of the enhancement of the cognitive reserve by music training and by the educational level can be related to previous works showing that the psychoacoustical improvements produced by music training are accompanied by neural plasticity changes in the auditory system (Herholz and Zatorre, 2012). Music related brain plasticity can be observed during early childhood, as evidenced by improved speech in noise perception and larger brainstem responses in children with music training (Strait et al., 2012). In addition, the effects of 8 weeks of auditory training are manifest in behavioral and in electrophysiological subcortical responses in older adults (Anderson et al., 2013). Notably, White-Schwoch et al. (2013) showed that the latencies of brainstem responses in aged individuals are faster in those with music training performed decades before, during childhood or adolescence. In the present work, we confirmed the protective consequences of previous music training in elderly and presbycusis subjects using a perceptual task. Importantly, these protective effects appears with 1 year of musical training, showing that it is not necessary to be a professional musician to get these protective effects. However, whether there is a greater protective effect with more years of music training or in professional musicians is a question that should be addressed in future studies. In addition, the lack of objective measurements of music training might be another limitation of the present results.

Regarding educational level, we found that in presbycusis patients, the years of education were important for MBEA performance in temporal tasks (T4 and T5), while music training for pitch tasks (T1, T2, and T3) and for meter task (T5), showing that different cognitive reserve factors can have consequences on different dimensions of music perception.

## Conclusion

Here, we give normative data for MBEA performance in a Latin-American population, showing age and educational effects. In addition, we demonstrate that music perception is impaired in symptomatic presbycusis subjects. The temporal and melodic impairments of presbycusis patients are diminished by the background educational level and music training.

## Author Contributions

PD, FM-G, FP, GV, and MR designed research. GV, MR, CM, RO, and FP performed research. FM-G, CD, AD-S, and PD analyzed data. FM-G, AD-S, CD, MR, and PD wrote the manuscript.

## Conflict of Interest Statement

The authors declare that the research was conducted in the absence of any commercial or financial relationships that could be construed as a potential conflict of interest.

## Footnotes

1 www.minsal.cl
